# Behavior of the Avian Parasite *Philornis downsi* (Diptera: Muscidae) in and Near Host Nests in the Galapagos Islands

**DOI:** 10.1007/s10905-021-09789-7

**Published:** 2021-11-17

**Authors:** Courtney L. Pike, Ismael E. Ramirez, David J. Anchundia, Birgit Fessl, George E. Heimpel, Charlotte E. Causton

**Affiliations:** 1 Charles Darwin Research Station, Charles Darwin Foundation, Puerto Ayora, Santa Cruz Ecuador; 2 Department of Behavioral and Cognitive Biology, University of Vienna, Vienna, Vienna Austria; 3 Department of Entomology, University of Minnesota, St. Paul, MN USA

**Keywords:** Insect behavior, Ectoparasite, Invasive species, Nest visitation, Life history, Host-parasite interactions

## Abstract

The Avian Vampire Fly, *Philornis downsi*, has invaded the Galapagos Islands, where it causes high mortality of endemic and native landbird species, including most species of Darwin’s finches. Control methods are under development, but key information is missing about the reproductive biology of *P. downsi* and the behavior of flies in and near nests of their hosts. We used external and internal nest cameras to record the behavior of *P. downsi* adults within and outside nests of the Galapagos Flycatcher, *Myiarchus magnirostris*, throughout all stages of the nesting cycle. These recordings showed that *P. downsi* visited flycatcher nests throughout the day with higher fly activity during the nestling phase during vespertine hours. The observations also revealed that multiple *P. downsi* individuals can visit nests concurrently, and that there are some interactions among these flies within the nest. Fly visitation to nests occurred significantly more often while parent birds were away from the nest than in the nest, and this timing appears to be a strategy to avoid predation by parent birds. We report fly mating behavior outside the nest but not in the nest cavity. We discuss the relevance of these findings for the adaptive forces shaping *P. downsi* life history strategies as well as rearing and control measures.

**Supplementary Information:**

The online version contains supplementary material available at 10.1007/s10905-021-09789-7.

## Introduction

Island ecosystems are increasingly challenged by invasive species that have been introduced by humans (Simberloff 2010; Bellard et al. 2017; Spatz et al. 2017; Lenzner et al. 2020). These invasive species threaten the health and survival of often naïve endemic species through interactions such as predation, competition, parasitism, or as vectors of parasites and pathogens (Causton et al. 2006; Reaser et al. 2007; Blackburn and Ewen 2016; Russel et al. 2017). While there is extensive research on the impacts attributed to alien plants and vertebrates (Medina et al. 2011; Pyšek et al. 2012; Spatz et al. 2017), less is known about introduced parasites and how they interact with biodiversity in these novel environments and what makes them successful invaders (Poulin 2017).

At least 499 species of insects have been introduced to and established in the Galapagos Islands, including 35 species that are parasitic on animals or plants, and additional surveys will likely reveal more (Toral-Granda et al. 2017). One of the most devastating introduced insects is the Avian Vampire Fly, *Philornis downsi*, a muscid whose larvae are ectoparasites of nestling birds (Fessl and Tebbich 2002). While the first report of *P. downsi* in bird nests occurred in 1997 (Fessl et al. 2001), records in museums date back to the 1960 s (Causton et al. 2006; Fessl et al. 2018). This fly is native to mainland South America and Trinidad (Bulgarella et al. 2015; Fessl et al. 2018; Koop et al. 2020), but it now also has a widespread distribution and broad host range in Galapagos, infesting nests of at least 21 small landbird species (Fessl, et al. 2018; Anchundia and Fessl 2020; Coloma et al. 2020). The introduction of this parasite has been highly detrimental to the reproductive success of small Galapagos landbirds, including various species of Darwin’s finches (Kleindorfer and Dudaniec 2016; Koop et al. 2016; Fessl et al. 2018; McNew and Clayton 2018). Indeed, *P. downsi* is now regarded as a leading causal factor in the observed population declines of passerine species in Galapagos, and, as such, it is a priority for conservation organizations to develop tools for its management (Cunninghame et al. 2012; Causton et al. 2013). Control options that are being evaluated include insecticidal treatment of nests and trapping as stop-gap methods and biological control and the Sterile Insect Technique as long-term options (Knutie et al. 2014; Heimpel 2017; Bulgarella et al. 2017; Fessl et al. 2018; Boulton et al. 2019; Bulgarella et al. 2020; Ramirez et al. Accepted).

The behaviors exhibited by *P. downsi* in and around nests of its hosts have implications for the development of control strategies for this fly and prior studies have provided information on nest-associated behaviors and interactions. Video recordings from within the nests of three species of Darwin’s finches from 2008 revealed that adult *P. downsi* visit nests during the incubation and nestling phases of bird development (both during the day and night), and may typically occur when adult birds are absent (O’Connor et al. 2010). These observations also revealed that adult *P. downsi* tend to oviposit on nest material near nestlings and that larval feeding is primarily nocturnal. Other observations of *P. downsi* behavior outside of an active nest of the Galapagos Flycatcher, *Myiarchus magnirostris*, showed that adult flies landed outside the nest and walked into it once a parent flycatcher had exited the nest (Lincango et al. 2015). Some use of defense strategies by parents or nestlings against *P. downsi* has been documented in Darwin’s finches, including preening of *P. downsi* larvae by nestlings or female parents, and antibody production against *P*. *downsi* by female parents (Huber et al. 2010). However, these appear to do little to protect nestlings from larval feeding by this parasite (O’Connor et al. 2010; Koop et al. 2013).

Despite these advances, there are still considerable gaps in our knowledge about how and when *P. downsi* adults locate and interact with their bird hosts. Furthermore, nothing is known about the courtship and mating behavior of *P. downsi*. In this study, we observed active nests of the Galapagos Flycatcher in artificial structures as a means of obtaining additional information on the behavior of *P*. *downsi* and its host inside and outside host nests. We also provide the first data on the interactions between *P. downsi* and its host over the entire bird developmental cycle – from incubation to fledging. Our main aims were to determine (i) if *P*. *downsi* preferentially visits host nests at certain times of the day or night and during certain stages of the reproductive cycle of birds, (ii) if multiple adult *P. downsi* can be found in host nests at the same time and if there are any interactions among them, (iii) which factors influence nest visitation by *P*. *downsi*, (iv) if Galapagos Flycatcher hosts exhibit defense strategies against *P*. *downsi* parasitism, and (v) if host nests are used by *P. downsi* as mating rendezvous sites. Additionally, we aimed to identify any natural enemies of *P*. *downsi* that might be present within host nests. Addressing these questions will aid researchers in developing techniques for controlling this fly, including techniques that could disrupt host or mate finding. This information will also aid efforts underway to develop a laboratory rearing system for *P. downsi*, which is needed to evaluate control methods (Lahuatte et al. 2016; Sage et al. 2018), in particular how to stimulate flies to mate and lay eggs.

## Methods

### Study Site and Host Species

This study was conducted in the arid zone of the southern part of Santa Cruz Island, Galapagos, Ecuador. The field site, El Barranco (0^°^ 44’ 14.0’’ S; 90^°^ 18’ 4.1’’ W, ~8 m (m) above sea level), is located in the Galapagos National Park, adjacent to the Charles Darwin Research Station (CDRS). This site is a deciduous dry forest, consisting of *Opuntia* and *Jasminocereus* cacti, palo santo (*Bursera graveolens*) and *Acacia spp.* trees, and a variety of herbaceous shrubs (Hamann 2011).

The Galapagos Flycatcher is widespread throughout Galapagos (Harris 1973) and is susceptible to *P. downsi* parasitism (Fessl and Tebbich 2002; Lincango et al. 2015). The incubation and nestling phases of the Galapagos Flycatcher are generally between 14 and 17 days each (C. Pike, unpublished data). This species is the only Galapagos landbird that readily nests in artificial structures (Lanyon 1978; Ervin 1994), including bamboo towers with cavities that have been constructed to study the biology of this bird and that were used in this study. The bamboo nest towers (3 m tall x 20-35 cm wide), each contained 3 to 11 cavities with entrances that measured approximately 10.5 cm high x 7.5 cm wide and nest boxes made of wood (25 cm high x 16 cm x 16 cm) with one entrance hole (3.75 cm radius).

### Video Recording

**Fig. 1:**
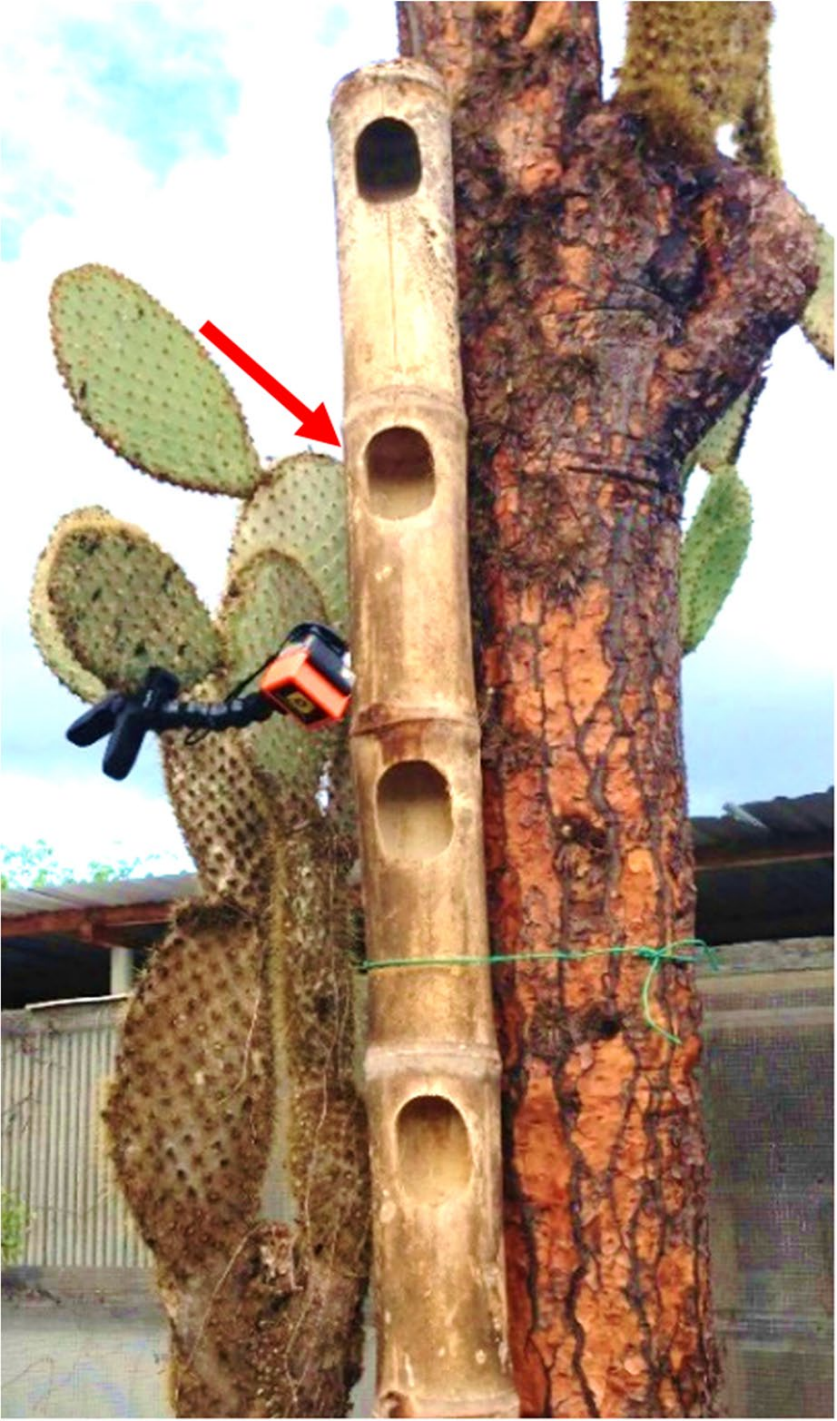
External video camera set-up for filming a nest in a bamboo tower with an extended-life battery, protective casing and flexible clamp mount facing the entrance of an active Galapagos Flycatcher nest. Arrow indicates active nest cavity. Photo: I. Ramirez

We filmed interactions between the Galapagos Flycatcher and *P. downsi* in three active nests built in three of the artificial bamboo cavities (two in 2015 and one in 2016). Filming of the first nest began on 23 March 2015. The three nestlings in the nest were estimated to be four days old (see below). The nest was filmed for 12 days - one day after all nestlings had fledged (Table 1). Filming of the second nest began on 3 June 2015. This nest contained three nestlings that were estimated to be seven days old. This nest was filmed for six days; the nestlings were found dead after the sixth day (Table 1). The third nest was filmed from 8 January to 20 February 2016. This nest contained four eggs and was estimated to be at day three of the incubation phase at the beginning of filming. Filming continued from this day until 17 days after fledglings had left the nest, a total of 47 days (Table 1). The three nests were filmed externally, focusing on the entrance of the nest and surrounding area, using a GoPro Hero 3+ camera (GoPro Inc., San Mateo, CA, USA). Filming was generally continuous between 6:00 and 18:30 with batteries and SD cards replaced once daily at noon (see Supplementary Table 1). The cameras were secured to a nearby *Opuntia* tree or to the bamboo tower with a flexible clamp mount with the camera aimed at the opening of the nest (Fig. 1). The cameras were 30-50 cm away from the entrance, allowing free entrance and exit of the flycatcher parents and additionally enabling the filming of *P. downsi* flight activity near the nest.

The nest studied in 2016 was also filmed from inside the nest cavity using an infrared nest box spy camera (3.5 mm lens, Bunker Hill Security), which ran for 24 h/day. The camera was positioned on the ceiling inside of the bamboo cavity at 27 cm from the base of the nest and approximately 25-26 cm from the nest material, to include most of the nest material in camera view. The nest camera was connected to a DVR Receiver (Spy Camera Cctv.com Model W720, Camarillo, CA, USA). Twelve-Volt sealed lead batteries and solar panels were used to provide energy continually. Internal recordings were made based upon a motion detection setting controlled by the DVR in which clips including approximately five seconds of filming before the initiation of movement were incorporated into a saved clip that continued until the motion stopped. Pilot tests showed that the video camera was sensitive enough to be triggered by a fly walking into view, a gecko entering the nest, and nestlings moving within the nest. The internal nest camera recorded activity from 9 January to 23 February, 2016. This included 17 days post-fledging to record the behavior of flies emerging from pupae (Table 1, Supplementary Table 2).

### Video Analysis

The external camera video recordings collected in 2015 were viewed and analyzed using Windows Media Player by one observer. The external and internal nest camera video recordings from 2016 were analyzed using the BORIS behavioral software program (Friard and Gamba 2016, v. 2.81) by multiple observers. This program was used to standardize observations and minimize observer bias from multiple observers. Flies observed within the nests were characterized as either (i) extending their ovipositors, (ii) engaging in other oviposition-like behavior, or (iii) doing neither of these things.

### Confirmation of Fly Identification

The video images were of insufficient quality to identify flies definitively as *P. downsi*. For this reason, we conducted supplementary studies during 2017 – 2019 to identify flies entering Galapagos Flycatcher nests. We captured flies visiting four Galapagos Flycatcher nests in three bamboo towers and one nest box for species identification during 14-18 April 2017 (nest 1), 21-30 April 2017 (nest 2), 13-23 March 2018 (nest 3), and 18-20 March 2019 (nest 4), from 16:30 to 18:10. Nests 1 and 2 were in the nestling stage, while nest 3 was in the incubation phase. Nest 4 was also in the incubation phase on the first day of fly trapping, but with newborn nestlings the following two trapping days. The four nests were monitored for 85 - 90 min per day for 26 days in total (11 days during the incubation period and 15 days during the nestling phase). We trapped the flies after they entered the nest cavity (when birds were not present), by placing a custom-made flytrap or a large Ziploc bag (26.8 × 27.3 cm) over the nest entrance. The custom flytrap consisted of a modified insect cage (30 × 30 cm) with a 10 cm diameter hole in one side, which was surrounded with part of a bicycle inner tube to ensure that the cage entrance fit securely against the entrance in the bamboo. Following trapping, each fly was identified to species following Couri (1999). In total, one individual was caught during the incubation phase and eight individuals during the nestling phase. All flies were identified as female *P. downsi*.

### Nest Check and Nest Collection

Nests of the Galapagos Flycatcher were observed every morning for activity of parent birds. If a nest did not exhibit activity for two hours, it was checked using an endoscopic fiber-optic camera with a wireless monitor (dnt Findoo, shaft 17 mm diameter, fiber-optic cable length 91 cm) mounted on a pole to determine the status of the nest. Final nest status was categorized as “fledged,” when the nest no longer contained nestlings and fledglings were seen outside the nest or “with dead nestlings,” when only dead nestlings were found in the nest. In 2015, once each nest was determined to be inactive, it was collected and inspected at the CDRS *P*. *downsi* laboratory the same day of collection to quantify *P. downsi* larvae and puparia. The larvae were categorized as first, second or third instars based upon the size and spiracular slit morphology (Fessl et al. 2006b). We categorized pupal casings (puparia) as either emerged, unemerged, or parasitized by Hymenoptera. In 2016, filming continued for 17 days post-fledging after which the nest was inspected in the CDRS laboratory.

### Statistical Analyses

We used generalized linear mixed models (GLMMs) using *lme4* in RStudio (R Core Team 2013) with Poisson error structure to test whether the number of *P. downsi* nest visits per hour (the dependent variable) during the nestling phase was significantly affected by (i) the time of day, (ii) the amount of time adult birds spent outside the nest per hour, and (iii) the number of times the adult birds visited the nest per hour, as fixed variables Nest identity was coded as a random effect and the number of observations was added as a second random effect as well to account for overdispersion of the data. Additionally, we tested for correlations between the continuous GLMM predictors using linear regressions.

We used a two-tailed log-likelihood goodness-of-fit (G) test with William’s correction factor (Sokal and Rohlf 1981) to determine whether the presence of parent flycatchers within the nest affected the propensity of *P. downsi* flies to enter the nest cavity. This test was done separately for nests in the incubation and nestling phases and observed proportions were compared to expected, or null, proportions. Under the null hypothesis, *P. downsi* visits are assumed equally likely when adult birds are present within the nest or not. We calculated the expected number of *P. downsi* visits under each condition based on the proportion of time adult birds spent in or out of the nest (27 % and 73 % of time, respectively, pooled for the nestling phase from all three nests, and 40 % and 60 %, respectively, for the incubation phase of the nest filmed in 2016). We also used a Kruskal-Wallis test to test whether the time a fly spent in the nest differed significantly between phases (incubation, nestling, and post-fledging phases). We used XLSTAT in Microsoft Excel (2016) to conduct these analyses. Lastly, we used a Chi- square contingency table test in RStudio (R Core Team 2013) to determine whether instances of ovipositor extension or apparent oviposition behavior were more likely to be observed during the incubation or nestling phases.

## Results

### Galapagos Flycatcher Fledging Success and Parasite Load

Each filmed nest contained three or four nestlings. While all nestlings fledged successfully from the March 2015 and the 2016 nest, fledging success was zero in the June 2015 nest (Table 1). The three nestlings in this nest died during the last day of filming, when they were estimated between 11 and 13 days old. The number of *P. downsi* recovered from each of the three nests ranged from 87 to 135 (Table 1). Both larval and pupal stages of *P. downsi* were found in the nests from 2015, but only empty puparia were found in the 2016 nest, which was collected 17 days post-fledging (Table 1). We did not find any larvae or pupae of insect species other than *P. downsi* in the nests.

**Table 1:** Information on Galapagos Flycatcher nests filmed in 2015 (using external cameras) and in 2016 (using external and internal cameras)

Nest*	2015 March	2015 June	2016 Jan-Feb**
Video recordings			
Hours of recording (EX) Hours of recording (IN) Number of days filmed (EX) (IN)	169 (N) - 12 (N)	67 (N) - 6 (N)	78 (I)/ 165 (N)/ 142 (PF) 233 (I)/ 318 (N)/ 329 (PF) 12 (I)/18 (N) /14 (PF) 11 (I)/18 (N) /17 (PF)
Bird information			
Number of eggs	n/a	n/a	4
Number of nestlings	3	3	4
Number of dead nestlings Number of fledglings	0 3	3 0	0 4
Fly information			
Number of visits by flies to nest (EX)	44	107	9 (I)/ 161 (N)/ 6 (PF)
Mean fly visits per day filmed	3.70 ± SE 1.84	17.80 ± 2.86	0.75 ± 0.37 (I)/ 9.00 ± 2.53(N)/ 0.36 ± 0.23 (PF)
Highest number flies in nest at one time	1	5	11
Total P. downsi found in nest:	87	135	114
1st instar larvae	0	4	0
2nd instar larvae	6	13	0
3rd instar larvae^a^	2	38	0
Unemerged puparia^b^	77	77	0
Empty puparia	2	3	114

See Supplementary Tables 1 and 2 for a summary of the amount of time spent filming. * (EX) = external nest camera, (IN) = internal nest camera. ** (I) = Incubation phase, (N) = Nestling phase, (PF) = Post-fledging phase

^a^ Larvae pupated and emerged in laboratory setting

^b^ For the June 2016 nest, 18 puparia were parasitized with emergence hole sizes common to *Nasonia* (Hymenoptera: Pteromalidae) or *Exoristobia* (Hymenoptera: Encyrtidae) parasitoids

### Fly Visitation of Nests

**Fig. 2:**
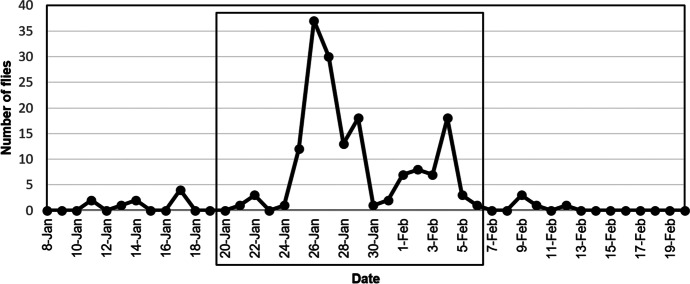
Fly visits per day over the reproductive cycle of a Galapagos Flycatcher nest filmed with an external camera in 2016. The box indicates dates when nestlings were present in the nest with dates to the left of the box indicating the incubating phase and areas to the right of the box indicating the post-fledging phase

**Fig. 3:**
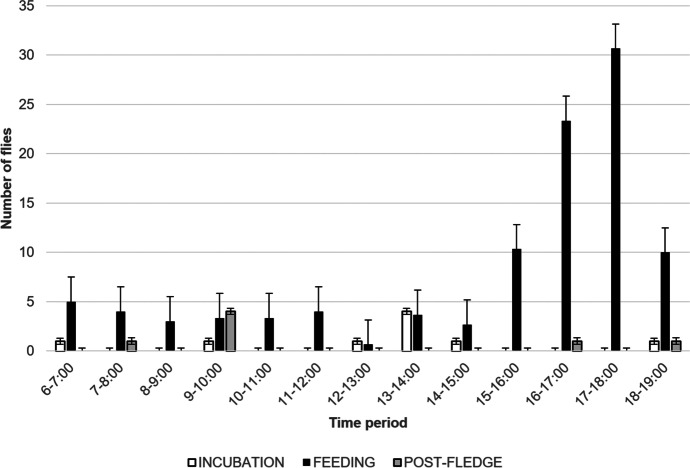
Fly visitation to Galapagos Flycatcher nests recorded at hourly intervals using an external camera. Values shown indicate the number of flies that entered nest cavities per hour filmed +/- Standard Error. For the nestling phase, the number of flies entering are hourly averages from the three nests combined

Fly visits were recorded during all three nesting phases of the 2016 nest, with 0.12 visits/ hour filmed during incubation, 0.98 visits/ hour filmed (91 % of total visits) during the nestling phase, and 0.04 visits/ hour filmed in the post fledging phase (see Supplementary Table 1 for total hours of filming). For this nest, peak visitation occurred when the nestlings were six days old (2.94 fly visits/ hour filmed, 37 visits, Fig. 2). For the nests filmed in 2015 during the nestling phase only, peak visitation occurred when the nestlings were nine days old (0.82 fly visits/ hour filmed, 10 visits) and eight days old (2.40 fly visits/hour filmed, 29 visits), in March and June respectively. Additionally, no flies were seen entering the June nest on the last day of filming, during which the nestlings died. Both the time of day and the presence of parent birds had effects on *P*. *downsi* nest visitation behaviors. Most visits during the nestling phase occurred in the afternoon, with peak visitation by flies between 17:00 and 18:00 h. Trends for visitation times during the incubation or post-fledging phases were not discernable because of the low number of fly visits (both phases) combined with fewer hours of video recording (incubation phase; see Supplemental Table 1) (Fig. 3). No nocturnal visits were recorded by the internal nest camera.

**Fig. 4:**
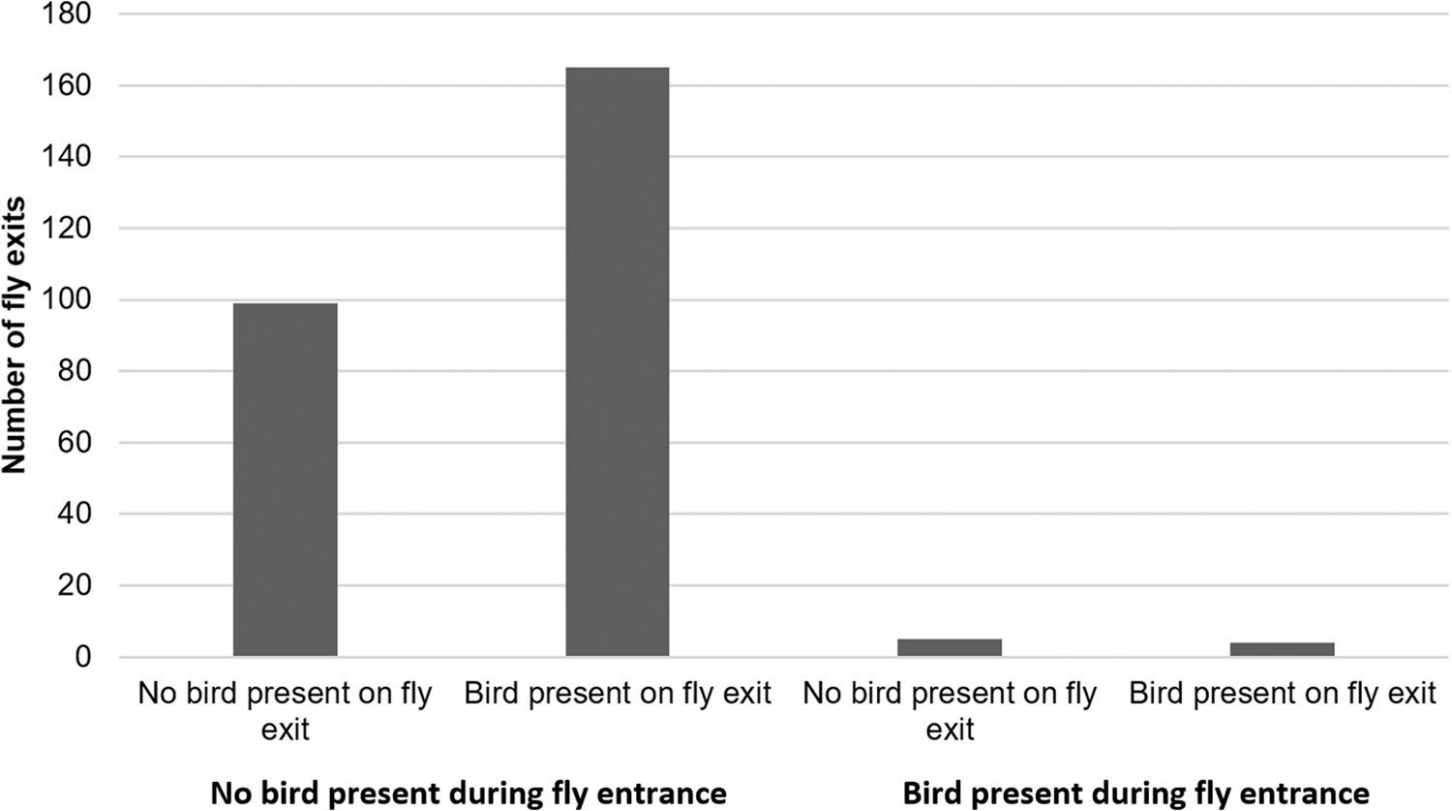
Number of fly entrances and exits when compared to bird presence and absence at the nests. Data from the three nests combined (two filmed in 2015 during the nestling phase and one filmed in 2016 during the incubating and nestling phases combined)

The GLMM on fly visitation (visits/hour) during the nestling phase showed a highly significant effect of time of day and marginally significant effects for both the number of adult bird visits per hour and the time they spent outside the nest per hour (Table 2). In addition, the Goodness-of-Fit test showed that the fly visitation rate (visits/hour) was significantly higher when parent birds were absent from the nest during the nestling phase (G = 122.86, d.f. = 1, p < 0.0001, only nine of 312 flies entered while the parent bird was inside the nest cavity; Fig. 4). There was no significant correlation between time of day and the number of adult bird visits (F_1,209_ = 0.64, *P* = 0.425) or the time that birds spent outside of the nest (F_1,209_ = 1.28, *P* = 0.259), which suggests that different bird behavior over the course of the day did not drive the results of bird behavior affecting fly visitation rates.

The timing of fly entrance into nests once flies had landed adjacent to nests was associated with the behavior of parent birds as well. During the incubation phase, flies landed near the nest on average 1.76 ± 0.42 min. after the parent bird had left (n = 8), and flies entered the nest cavity on average 0.22 ± 0.05 min. after arrival (n = 9). These values were 1.45 ± 0.11 min. (n = 318) and 0.17 ± 0.01 min. (n = 307) for the nestling phase. Flies normally walked into the nest cavity (95 % of all fly entrances (n = 327)), but exited the nest cavity flying (94 % of all exits (n = 318)). Flies that landed but did not enter the nest (n = 68 observations) flew away less than five seconds after landing. The average time spent in the nest cavity did not significantly differ between nest phases (H = 2.771, d.f. = 2, p = 0.25; incubation phase 1.78 ± 0.54 min. (n = 9); nestling phase 1.38 ± 0.12 min. (n = 271); post fledgling stage 1.14 ± 0.65 min. (n = 6)). Lastly, four of the six visits to the nest during the post-fledging phase took place on the same day that fly emergence from puparia occurred (on days three and day six post-fledging).

**Table 2:** Results of a generalized linear mixed model assessing the effects of time of day, the amount of time parent birds spent away from the nest per hour, and the number of bird visits to the nest per hour on the rate of *P. downsi* visitation (also per hour) during the nestling phase, with nest identity and the number of observations per nest as random effects

	Estimate	Std. Error	*Z*	*P*
(Intercept)	-3.64255	0.70271	-5.184	2.18e-07 ***
Time of day	0.20631	0.02812	7.336	2.21e-13 ***
Time bird away from nest	-0.05739	0.03070	-1.869	0.0616
Number of bird visits to nest	0.06334	0.03296	1.921	0.0547

The analysis included data from all three nests

While the majority of observations using the external camera involved a single *P. downsi* adult visiting a nest at a time, there were some instances of multiple adult flies being present in a nest at the same time. Four events in which between two and five flies were registered in a nest cavity simultaneously were recorded during the nestling phase from the June 2015 nest. In the 2016 nest, multiple flies were also recorded in the nest cavity at the same time, with a maximum of 11 flies recorded on day seven of the nestling phase (Table 1).

### Fly Activity Within the Nest

Adult flies were observed walking on flycatcher eggs and nestlings as well as on various substrates within the nesting cavity. During the incubation phase, adult *P. downsi* spent up to 73.5 % of the time observed on eggs. Flies that walked on host eggs spent a mean of 15.4 ± 2.08 s. (n = 27, max 33 s.) on eggs per observation. Flies were only seen walking across or standing on eggs; no instances of flies noticeably palpating them with their mouthparts were observed. During the nestling phase, adult flies were observed walking on nestlings of ages ranging from less than one day to fifteen days old (two days before fledging). The flies also walked on the nest base underneath the nestlings’ bodies while the nestlings were sitting and standing. No visible reaction by the nestlings to the flies was observed.

We did not observe flies ovipositing on eggs or nestlings, but noted behaviors that suggested that flies were laying eggs in the nesting material; such as ovipositor extension or, “oviposition-like behavior,” in which the abdomen was pointed down towards the nest material and a bobbing movement anteriorly - posteriorly was noted. Ovipositor extension or oviposition-like behavior was observed in 76 % of the observations of *P. downsi* in the incubation phase (n = 25) and 71 % of observations during the nestling phase (n = 248). No significant differences were found between the incubation and nestling phases for the proportion of times *P. downsi* adults exhibited this behavior (χ² = 1.19, d.f. = 2, p = 0.5505).

We recorded 17 instances of multiple flies present in the nest cavity using the internal nest camera, all from nests with 4-9 day old nestlings and in six of these cases, interactions between flies were observed. Three of these interactions were particularly noTable In one of these interactions, one fly encountered a second fly that appeared to be in the process of ovipositing; the second fly responded to the first fly by pushing it with its legs. In another interaction, one fly appeared to oviposit, then the second fly walked toward it, and the first fly walked forward ~ 2 mm. The first fly again appeared to initiate oviposition, but the second fly approached it again, pushing it with its legs. The second fly then appeared to oviposit in the location of the first fly’s apparent oviposition attempt. In a third interaction, one fly walked into a second fly and the second fly walked forward. Then, both appeared to oviposit within 1-2 cm of each other.

### Larval Activity in the Nest

*Philornis downsi* larvae (first, second and third-instars) were first seen via the internal nest camera when the nestlings were five days old, two days after the parent stopped staying in the nest overnight. From then on, adult flycatcher visits to the nest overlapped with larval activity only from 17:40 until dark (~18:40) and in the mornings from sunrise (~05:20) until 07:00. On day six of the nestling phase, early instar larvae were seen protruding from the nasal cavities and ear canals of two nestlings and the first large second or third instars were seen in the nest base, near the nestlings. By day seven, many larvae were seen on the surface and the sides of the nest at night. Throughout the nestling phase, larvae were observed on multiple body parts, including within the nares (35.0 %) or ear canals (2.5 %), or externally on the wing (22.5 %), head and neck (17.5 %) or back, abdomen or chest (22.5 %) of the nestlings. The longest time a larva was seen attached to a nestling was 101 min. on the back of the head of a nine-day old nestling. This appeared to be a third instar larva. When the larva detached, the nestling had a visible lesion. Mature larvae were observed with part of their bodies emerging from the nest material during nighttime hours for six days after the nestlings fledged.

### Fly Predation and Removal

Both external and internal cameras recorded instances of *P. downsi* predation and removal. External cameras revealed adult Galapagos Flycatchers preying on flies outside or near the nest entrance four times during the nestling stage (once in 2015 and three times in 2016). On all occasions, the bird was arriving or standing on the outside of the nest cavity and successfully caught and either ate the fly or fed it to a nestling. In one of these events, the fly was standing on the outside of the bamboo, while in all other instances the fly was exiting the cavity and was in flight when it was caught. The internal nest camera recorded multiple occasions in which adult birds were observed pecking at the nest material and eating unidentified objects, but we could only once confirm that a *P. downsi* larva was taken in this way. Additionally, in one instance, an adult Galapagos Flycatcher was observed pecking at the nostril of a nestling, but the adult did not appear to remove anything. The removal of fly larvae by nestlings themselves via flapping and preening was observed during the hours 1:00-4:00 and 18:00-21:30 when nestlings were 9, 10, and 14 days old but it was not clear that these preening behaviors were acts of predation. Additionally, nine to 13 day old nestlings used their feet to ‘scratch’ their heads during the night (20:00-01:00) and morning (~7:45) hours but this behavior did not necessarily remove larvae.

In 2016, we observed geckos inside the nest cavity during incubation (five times), nestling development (13 times), and after fledging (24 times). These were all identified as the introduced Mourning Gecko, *Lepidodactylus lugubris* and may have represented repeat visits by the same individuals or multiple individuals. Gecko visits occurred only between 18:39 and 05:35. Over this period, six successful predation events on *P. downsi* larvae were observed, all of which took place after nestlings had fledged.

### Fly Emergence from Nest Material and Mating Behavior

**Fig. 5:**
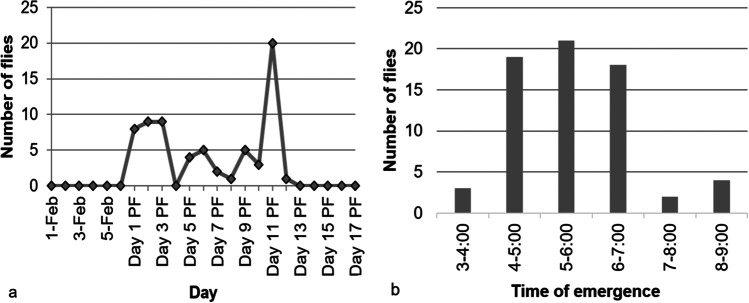
Number of *P. downsi* adult flies emerging from material of a Galapagos Flycatcher nest filmed with an internal nest camera in 2016 (**a**) per day and (**b**) by time period. PF indicates post-fledging phase. *P. downsi* was not seen emerging outside of the times displayed

Adult *P. downsi* emerged from their puparia within the nest material over a period of 11 days, starting one day after the nestlings fledged. An average of 6.09 ± 1.65 flies emerged per day with a maximum of 20 flies emerging on day 11 post-fledging (Fig. 5a); all recorded fly emergences took place between 03:00 and 09:00 (Fig. 5b). Although we found 114 open puparia in the nest material, we only recorded 67 instances of fly emergence, potentially due to the internal camera malfunctioning 18 % of the time during the post-fledging phase (see Supplementary Table 2). Thirty flies were registered exiting the nest cavity by the external camera during this time period. The remaining flies may have exited either during periods of the day when the camera failed (see Supplementary Table 1) or during nighttime hours when the camera was not on. Flies exited the nest cavity between 06:48 and 13:47 with the highest number of 19 flies exiting the nest cavity between 07:00 and 09:00. By comparing video recording from the internal and external nest cameras, we found that some flies stayed in the nest cavity after emergence, in one case for up to 11 h.

Behaviors that appeared to be mating by presumed *P. downsi* flies were observed outside the nest cavity on four occasions during the post-fledging phase in the 2016 nest. The first of these mating events occurred on the day that the last fly emergence was recorded, one day after the highest number of flies (20) was recorded emerging from the nest. The remaining three mating events took place two days after this. No flies were filmed exiting the nests on these days. The first mating event occurred 12 days post-fledging, at 17:35, and began when a fly landed just below the nest cavity. This was followed by a second fly landing nearby, at which point the first fly (the “resident” fly) walked toward the arriving fly. The two flies then walked in circles following each other before the resident fly mounted the arriving fly for ~ 42 s. After this, the flies backed away from each other, and the arriving fly flew away. The second event took place 14 days post-fledging, at 17:59 when a fly landed a few inches below another fly that was positioned just beneath the entrance to the nest cavity. Wing fanning by both individuals was observed, and the fly that had been present earlier (the “resident” fly) moved toward the arriving fly and mounted it for six sec. This was followed by the flies chasing each other for 35 s., after which the fly that had been mounted flew away. The third event occurred at the same time as the second event. A fly landed above the nest cavity and was approached by another fly already present on the bamboo. This resident fly appeared to mount the arriving fly for seven sec., after which the arriving fly flew away. The fourth instance also occurred 14 days post-fledging at 18:17. One fly was standing ~16 cm below the nest entrance, when a second fly landed approximately 2 cm away from the resident fly. The resident fly fanned its wings and walked to and mounted the arriving fly for 34 s. After this, the arriving fly flew away, while the resident fly stayed on the bamboo. A number of consistencies can be discerned from these four instances of attempted or actual mating. First, they all occurred around dusk. Second, mating appears to have been initiated by the resident rather than the arriving fly. Third, mounting was short, never exceeding one minute.

## Discussion

Video recordings taken outside and within nests of the Galapagos Flycatcher that were heavily infested with *P. downsi* have provided new insights into the behavior of this invasive parasitic fly, though results should be interpreted with some caution given the low sample size (three nests) and the limited data collection period for two nests. By tracking three nests during the nestling phase and monitoring fly activity from the onset of the incubation phase to post-fledging in one of these, we determined that *P. downsi* visitation of nests appears to be highly dependent on the time of the day and is influenced by the presence of adult birds; when birds are in the nest, flies rest outside the nest until the birds leave. This suggests an adaptation of *P*. *downsi* to nesting behaviors of its hosts. The highest visitation rates of *P. downsi* to bird nests were during the nestling phase during late afternoon/ dusk. Nests were visited by high numbers of flies (up to 37 visits occurred during daylight hours in a single day) with multiple individuals (up to 11) in the nest cavity at the same time. Larvae were observed feeding on all parts of the nestlings, however, active late-instar larvae were also observed six days after the nestlings had fledged, suggesting that larvae can survive for long periods without a live host before pupating. While no mating was observed inside the nest cavity, observations suggest that *P. downsi* rendezvous to mate outside the nest cavity. Lastly, low level parasitism of puparia by parasitoid Hymenoptera and predation of *P. downsi* larvae by introduced geckos were recorded, which, in addition to fly captures by adult Galapagos Flycatchers provides some mortality of *P. downsi*, but likely not enough to reduce numbers substantially.

Although we could not categorically confirm that all flies observed in our recordings were *P. downsi*, the evidence collected in this and prior studies strongly supports the hypothesis that they were indeed *P. downsi* for the following reasons: (1) We did not find larvae or pupae of other Diptera in the nests filmed in our study. This suggests that flies of other species were not ovipositing in the nest material; (2) Muscid larvae and pupae have not been reported in the more than 1500 + nests that have been inspected in the last eight years by scientists (P. Lahuatte, pers. comm) or in the literature - a prior report of an unidentified muscid in nests (Fessl and Tebbich 2002) was later confirmed by B.F. to be *P*. *downsi*; (3) The only other flies that have been found in nests other than *P*. *downsi*, are sarcophagids (Fessl et al. 2006a). These species are typically associated with nests containing dead nestlings - two of our nests (March 2015 and 2016 nests) did not contain dead nestlings; the third nest did not contain dead nestlings during recorded fly visitation, as no flies visited the last day of filming when the nestlings died; (4) Trapping of flies entering bird nests in 2017-2019 led to captures of only *P*. *downsi* and these flies exhibited similar behaviors to those filmed in 2015-2016.

Most observed fly visits took place during the nestling phase in the last hour of daylight, with the highest number of fly visits occurring in the middle of the nestling phase, when the nestlings were six to nine days old. This suggests that fly visitation may be influenced by the age of nestlings rather than other factors, such as weather. Young (1993) found the highest number of *P. carinatus* larvae on nestlings between six to nine days old, similar to our findings, although he suggested that *P*. *carinatus* and other bird-parasitic flies do not time egg laying to nestling age. The vespertine activity of *P*. *downsi* (i.e. activity during late afternoon/dusk, but not at dawn), on the other hand, could be explained by several adaptive hypotheses, including seeking optimal abiotic conditions, tracking resource availability and/or gaining safety from diurnal predators, such as parent birds. *Philornis downsi* may be active during dusk to avoid higher temperatures, as found in other insects (May 1979), or their evening activity may be correlated with lower light intensity, as shown in other species (Rieger et al. 2007; De et al. 2013). Alternatively, *P*. *downsi* may visit nests more often at dusk due to a genetic and/or hormonal-based internal rhythm related to egg laying (as reviewed in Howlader and Sharma 2006). The absence of activity at nests during dawn, which is also characterized by cooler temperatures, could reflect a prioritization for seeking food resources such as nectar, pollen or plant exudates (see Fessl et al. 2018) during morning hours before they have dried up or been consumed by competitors. Lastly, while nighttime visits to nests would appear to be advantageous from the standpoints of both temperature and safety from parent birds (in our study adults stopped brooding at night when nestlings were three days old), we did not record *P. downsi* visiting nests at night as found by O’Connor et al. (2010).

*Philornis downsi* adults were more likely to enter nest cavities when adult birds were absent. This suggests that *P. downsi* can discriminate nests with and without adult birds and tend to avoid the former, thus reducing the risk of predation. In addition, fly visitation during the nestling phase was negatively correlated with long absences of adult birds from the nests and positively correlated with the number of bird visits to the nest. Both of these results suggests that the presence of adult bird activity in or near nests attracts *P. downsi* to the vicinity of nests. We therefore propose that *P. downsi* adults use cues (noise, odor, movement or infrared radiation) associated with the presence of adult birds to locate active nests while avoiding predation. This includes detecting a nest and waiting outside of it until a parent bird exits for the fly itself to enter, which was a common behavior of the flies recorded in our study. The few predation events that we observed occurred when a fly entered a nest while an adult bird was present or when a fly exited a nest while a bird had just landed at the nest entrance. The rarity of these events is consistent with predation avoidance by *P. downsi*.

Evidence of female fly visitation was recorded by the internal nest camera and allowed for descriptions of oviposition behavior by *P. downsi*. Observations of extended ovipositors and apparent oviposition behavior during the incubation and nestling phases suggest that at least 70 % of the flies recorded in the nest camera were female; however, it must be noted that some of the recorded events may have been individual flies re-entering the camera viewing area. Additionally, up to three confirmed female flies (with ovipositors extended) were seen in a nest at one time, which is consistent with previous population genetic studies indicating that multiple *P. downsi* females oviposit per nest (Dudaniec et al. 2010), and provides new evidence that oviposition events by several flies can take place simultaneously. Evidence of female flies in the nest cavity during the incubation phase supports prior reports of *P*. *downsi* oviposition during the incubation phase in nests of Darwin’s finches (Cimadom et al. 2016; Common et al. 2019; Cimadom and Tebbich 2020). On the other hand, observations of fly visitation and oviposition-like behavior during the late nestling phase concur with findings of first instar *P. downsi* larvae in host nests with nestlings close to fledging age (C. Pike, unpublished data; Kleindorfer et al. 2014). Oviposition at this state of bird development is somewhat paradoxical since fly eggs would appear to be deposited with insufficient time for completing larval development. However, these observations contribute to gathering evidence of such late oviposition by *P*. *downsi* females over the last decade, which may be a response to high population densities of flies and/or low availability of hosts (Kleindorfer et al. 2014; Causton et al. 2019; Cimadom and Tebbich 2020). As expected, there was no evidence for oviposition by visiting flies during the post-fledging phase and fly visitation was considerably lower in general. Given this, it is possible that the fly visits observed during the post-fledging phase could be either males or females seeking mates. Lastly, given that more visitation events were recorded than the total number of *P*. *downsi* parasites found in the nest, it may be that visiting females do not lay eggs during every nest visit. Alternatively, more *P*. *downsi* larvae may have been present, but did not survive to pupation due to larval competition, and/ or predation by birds or geckos.

We also documented times of activity and feeding sites for *P. downsi* larvae inside the nest cavity. Larval feeding took place from dusk to dawn; however, since this study we have also seen *P. downsi* larvae feeding on Galapagos Flycatcher nestlings during the day (personal obs., C.L.P.). We report larval feeding on most body parts of the nestlings, including on the head, neck, back, abdomen, and chest, congruent with prior reports of contusions on the abdomen and back of nestlings (e.g. Fessl et al. 2006b; Koop et al. 2011). Larval feeding mainly during the nighttime hours may be an adaptive strategy (Gold and Dahlsten 1989), as nestling activity is lower and Galapagos Flycatcher parents do not stay overnight after nestlings are approximately three days old.

By filming nests after nestlings departed, we were able to observe the timing of *P. downsi* emerging from pupae in the nest material. Multiple peaks of emergence may indicate multiple egg cohorts laid on different days, by one or multiple female flies, as reported in *P. carinatus* in Costa Rica (Young 1993) and consistent with reports of Dudaniec et al. (2010) for *P. downsi*. Additionally, our finding that adult flies emerged within host nests only during early morning hours suggests there may be an eclosion rhythm based on factors such as temperature or the circadian clock (Pittendrigh 1954), potentially providing an adaptive benefit, such as meeting potential mates, avoiding predation, and/or avoidance of desiccation (Cloudsley-Thompson 1960). Peak hours of adult eclosion from puparia have been previously reported in other avian nest parasites, including *Protocalliphora*, which took place during daylight hours, from 09:00-15:00 (Gold and Dalhsten 1989).

Insects commonly use hosts as rendezvous sites for mating (“resource-based mating,” Wilkinson and Johns 2005). We recorded four events of flies similar in size to *P. downsi* mating outside the nest cavity during the post-fledging phase around the time that flies were emerging from pupae. These events took place one to three days after the day that the highest numbers of flies were observed emerging from the nest material, but only the first event coincided with a known emergence event. Studies suggest that male *P*. *downsi* use pheromones to attract females (Collignon 2011; Mieles 2018). Given the ephemeral nature of host nests, typically available during the hot, wet season (January to April/May, Grant 1986), it may be that male *P*. *downsi* use nests to locate and attract females for mating. Since active nests may pose risks of predation by adult birds, nests in the post-fledging period out of which females are emerging may be a favored site for male pheromone release. Male courtship in flies that use resource-based mating strategies is generally minimal and can involve a repetitive action like wing waving, fanning (to disseminate pheromones) or wing vibrations (Burk 1981; Wilkinson and Johns 2005), such as was seen near the Flycatcher nest.

In our study, *P. downsi* density was high in all three nests and mortality imposed by natural enemies appeared to be minimal. There are currently seven parasitoid species (Hymenoptera) reported to sporadically use *P*. *downsi* as a host in the Galapagos Islands (Fessl et al. 2018). We found a small number of pupae that appeared to be parasitized by a member of the parasitoid families Pteromalidae or Encyrtidae, but we did not observe any parasitoids visiting the nest. Geckos were recorded preying upon *P. downsi* larvae, and were observed in all of the nest phases. Additionally, there was evidence of a low number of predation events by adult flycatchers. Furthermore, *P*. *downsi* individuals from the Galapagos Islands have been found to host endoparasites with unknown effects; however, further studies are required to determine whether they affect fly populations (Pike et al. 2020).

### Implications for Management

This study provides new information for researchers and conservationists attempting to control *P. downsi* in the Galapagos Islands and could also help to inform the management of other *Philornis* species that are affecting endangered bird species (Bulgarella et al. 2019). While this study focuses on only three nests, each nest provided a substantial amount of information and, importantly, showed consistent fly behaviors across years and nests. This suggests that similar behaviors occur at other nests of the same species. Additionally, while our study focused on the activity of *P. downsi* in and around Galapagos Flycatcher nests, it is probable that similar behaviors occur in association with nests of other Galapagos landbirds, including Darwin’s finches, in whose nests fly activity has been previously recorded (O’Connor et al. 2010; Koop et al. 2013). The observations on the timing of the nest visits during the reproductive cycle of the birds and the time of day visited have helped guide additional experimental studies on life history and control of *P. downsi*; for example, the timing of insecticide treatment in nests (Causton et al. 2019). This study also provides clues about the mating and courtship behaviors of *P. downsi*, which are critical for establishing captive colonies and developing methods such as sterile male releases (Lahuatte et al. 2016; Fessl et al. 2018). Using data from this study, mating and egg laying experiments may be timed more effectively, especially taking into consideration that the late afternoon is the time of day that *P*. *downsi* is most active at host nests.

Lastly, although interactions of *P. downsi* with the Galapagos Flycatcher could differ from interactions with other Galapagos birds that do not engage in predation while flying, the inferences regarding cue use for host finding might aid in directing future studies into how female *P. downsi* find their hosts. This, in turn, may lend to the development of management tools such as auditory, visual or olfactory decoys that could manipulate the fly’s behavior thus reducing parasitism pressure on at-risk bird populations (Sutherland 1998).

## Supplementary Information

Supplementary Table 1 (DOCX 16.3 KB)

Supplementary Table 2 (DOCX 14.6 KB)

## Data Availability

Data can be shared if requested.
