# Supplementary material for: Behavior of the Avian Parasite Philornis downsi (Diptera: Muscidae) in and Near Host Nests in the Galapagos Islands
Source: J Insect Behav. 2021 Nov 17;34(5-6):296–311. doi: 10.1007/s10905-021-09789-7 (PMC8813692; doi:10.1007/s10905-021-09789-7)
Supplement: Supplementary file 1 — (DOCX 16.3 KB) [file 10905_2021_9789_MOESM1_ESM.docx]

**Behavior of the avian parasite *Philornis downsi* (Diptera: Muscidae) in and near host nests in the Galapagos Islands**

**Journal of Insect Behavior**

**Authors**

Pike, Courtney L.^1,2*^, Ramirez, Ismael E. ^3^, Anchundia, David J. ^1,2^, Fessl, Birgit ^1^, Heimpel, George E. ^3^, Causton, Charlotte E.^1^

^1^ Charles Darwin Research Station, Charles Darwin Foundation, Santa Cruz, Galapagos Islands, Ecuador

^2^ Department of Behavioural Biology, University of Vienna, Althanstraße 14, 1090, Vienna, Austria

^3^ Department of Entomology, University of Minnesota, 219 Hodson Hall, St. Paul, MN 55108, USA

*Corresponding Author: [Courtney.L.Pike@gmail.com](mailto:Courtney.L.Pike@gmail.com)

**Supplementary Table 1:** Total video recording time, expressed as a percent from 6:00 to 19:00, for nests filmed externally in 2015 and 2016. For 2015, the calculation is based on the total number of days filmed during the nestling phase, not the entire phase as done for the 2016 nest.

|  | **2015 March** | **2015**  **June** | **2016**  **January-February** | | |
| --- | --- | --- | --- | --- | --- |
| **Time of day** | **Nestling phase**  **(12 days)**  **(% time)** | **Nestling phase**  **(6 days)**  **(% time)** | **Incubation phase**  **(12 days)**  **(% time)** | **Nestling**  **Phase**  **(18 days)**  **(% time)** | **Post-fledge phase**  **(14 days)**  **(% time)** |
| *6:00 – 7:00* | 82.6 | 77.8 | 14.6 | 48.2 | 55.8 |
| *7:00 – 8:00* | 83.3 | 100 | 23.6 | 82.7 | 100.0 |
| *8:00 – 9:00* | 83.3 | 100 | 66.7 | 92.2 | 99.4 |
| *9:00 – 10:00* | 83.3 | 100 | 83.3 | 99.8 | 92.9 |
| *10:00 – 11:00* | 83.3 | 100 | 77.9 | 100.0 | 89.2 |
| *11:00 – 12:00* | 72.2 | 97.2 | 64.5 | 96.0 | 80.6 |
| *12:00 – 13:00* | 55.6 | 81.9 | 60.6 | 85.5 | 67.5 |
| *13:00 – 14:00* | 83.3 | 77.8 | 58.3 | 71.9 | 44.9 |
| *14:00 – 15:00* | 100 | 91.7 | 58.3 | 63.7 | 50.4 |
| *15:00 – 16:00* | 100 | 91.7 | 58.3 | 62.8 | 68.9 |
| *16:00 – 17:00* | 100 | 91.7 | 40.3 | 65.0 | 71.6 |
| *17:00 – 18:00* | 100 | 91.7 | 25.0 | 61.1 | 66.1 |
| *18:00 – 19:00* | 87.5 | 83.3 | 20.1 | 43.3 | 38.8 |
| **Total % of phase filmed** | 85.4 | 91.5 | 50.1 | 74.8 | 71.2 |
