# Supplementary material for: Behavior of the Avian Parasite Philornis downsi (Diptera: Muscidae) in and Near Host Nests in the Galapagos Islands
Source: J Insect Behav. 2021 Nov 17;34(5-6):296–311. doi: 10.1007/s10905-021-09789-7 (PMC8813692; doi:10.1007/s10905-021-09789-7)
Supplement: Supplementary file 2 — (DOCX 14.6 KB) [file 10905_2021_9789_MOESM2_ESM.docx]

**Behavior of the avian parasite *Philornis downsi* (Diptera: Muscidae) in and near host nests in the Galapagos Islands**

**Journal of Insect Behavior**

**Authors**

Pike, Courtney L.^1,2*^, Ramirez, Ismael E. ^3^, Anchundia, David J. ^1,2^, Fessl, Birgit ^1^, Heimpel, George E. ^3^, Causton, Charlotte E.^1^

^1^ Charles Darwin Research Station, Charles Darwin Foundation, Santa Cruz, Galapagos Islands, Ecuador

^2^ Department of Behavioural Biology, University of Vienna, Althanstraße 14, 1090, Vienna, Austria

^3^ Department of Entomology, University of Minnesota, 219 Hodson Hall, St. Paul, MN 55108, USA

*Corresponding Author: [Courtney.L.Pike@gmail.com](mailto:Courtney.L.Pike@gmail.com)

**Supplementary Table 2:** Total video recording time, expressed as a percent from 0:00 to 24:00, for the nest filmed internally in 2016.

| **Time of day** | **Incubation phase**  **(11 days filmed)**  **(% time)** | **Nestling phase**  **(18 days filmed)**  **(% time)** | **Post-fledge phase**  **(17 days filmed)**  **(% time)** |
| --- | --- | --- | --- |
| *0:00 – 1:00* | 90.9 | 94.4 | 96.6 |
| *1:00 – 2:00* | 90.9 | 88.9 | 94.1 |
| *2:00 – 3:00* | 90.9 | 88.9 | 94.1 |
| *3:00 – 4:00* | 90.9 | 80.1 | 94.1 |
| *4:00 – 5:00* | 85.0 | 73.1 | 93.3 |
| *5:00 – 6:00* | 81.8 | 61.4 | 85.6 |
| *6:00 – 7:00* | 78.5 | 57.4 | 82.4 |
| *7:00 – 8:00* | 63.6 | 51.8 | 82.4 |
| *8:00 – 9:00* | 68.6 | 55.0 | 82.4 |
| *9:00 – 10:00* | 78.8 | 65.6 | 87.5 |
| *10:00 – 11:00* | 78.2 | 72.2 | 88.2 |
| *11:00 – 12:00* | 83.6 | 72.9 | 88.6 |
| *12:00 – 13:00* | 89.2 | 70.9 | 97.4 |
| *13:00 – 14:00* | 84.1 | 67.8 | 100 |
| *14:00 – 15:00* | 90.9 | 73.7 | 100 |
| *15:00 – 16:00* | 85.6 | 75.6 | 85.6 |
| *16:00 – 17:00* | 90.9 | 83.3 | 79.9 |
| *17:00 – 18:00* | 97.7 | 83.3 | 63.0 |
| *18:00 – 19:00* | 100 | 83.3 | 56.1 |
| *19:00 – 20:00* | 100 | 78.2 | 42.7 |
| *20:00 – 21:00* | 100 | 74.4 | 41.2 |
| *21:00 – 22:00* | 100 | 69.5 | 35.9 |
| *22:00 – 23:00* | 100 | 70.3 | 37.0 |
| *23:00 – 24:00* | 100 | 80.2 | 43.5 |
| **Total % of phase filmed** | 88.3 % | 73.9 % | 77.2 % |
